# Association between PAI-1 gene − 657 4G/5G polymorphism and preeclampsia in Egyptian women: a case-control study

**DOI:** 10.1186/s12884-025-07737-3

**Published:** 2025-05-26

**Authors:** Omali Y. El-khawaga, Afaf M. ElSaid, Hwyda Ahmed, Aliaa N. El-Daw, Hend A. Shalaby, Mohammed F. Al-azzawy, Manar refaat

**Affiliations:** 1 Biochemistry Division, Chemistry Department, Faculty of Science, Mansoura University, Mansoura, 35516 Egypt; 2 Department of Women and obstetrics, Faculty of Medicine, Mansoura University, Mansoura, 35516 Egypt; 3 Genetic Unit, Department of Pediatrics, Faculty of Medicine, Mansoura University, Mansoura, 35516 Egypt; 4 Department of Medical physics techniques, Al-Imam University College, Tikrit, Iraq

**Keywords:** Pre-eclampsia, Hypertension, Single-nucleotide polymorphisms

## Abstract

**Abstract:**

Pre-eclampsia constitutes a pregnancy disease characterized by hypertension and proteinuria, as well as dysregulation of the coagulation cascade and hypo fibrinolysis that affects many systems. The purpose of this study is to examine the connection between (-657 4G/5G PAI-1) and the danger of preeclampsia in Egyptian females. A case-control study included 140 Egyptian pregnant women with PE along with 100 normotensive women. The study includes extracting genomic DNA from blood, detecting SNP with ARMS-PCR, and assessing metabolic parameters with serum samples. The study discovered significant variations in PAI-1 5G/4G polymorphism between sufferers and the control group in general (*P* < 0.001), dominant (*P* = 0.004), and recessive models (*P* = 0.009). Pre-eclamptic women had lower SOD activity and GSH levels but higher MDA and GPx activities than the normotensive pregnant group.

**Conclusion:**

This study indicated that the − 657 4G/5G PAI-I mutation is substantially linked to the development of PE in Egyptian women.

## Introduction

Pregnancy is linked to the progressive development of a hypercoagulable condition, with a physiologic rise in clotting factors including von Willebrand factor and fibrinogen. A healthy pregnancy requires sufficient blood circulation to the implantation site. In particular, fibrinogen levels jump by 50%. Physiological changes intended to aid in post-delivery hemostasis may complicate anticoagulant management for pregnant women. Furthermore, greater amounts and actions of thrombin-activated fibrinolysis inhibitory agents, plasminogen activator inhibitor-1, as well as plasminogen activator inhibitor-2, prevent fibrinolysis [1]. Maternal clotting and fibrinolysis stabilize pregnancy, but thrombophilia deformities can develop, increasing the risk of pregnancy-related thromboembolism, preeclampsia, and fetal loss in women with these abnormalities [2]. Preeclampsia is a critical maternal health problem across the world, producing severe morbidity and mortality in both newborns and mothers, as well as strongly contributing to fetal preterm birth and long-term heart illness in mothers [3]. The International Society for the Research of Hypertension in Pregnancy (ISSHP) describes preeclampsia as the incidence of abrupt hypertension, proteinuria, or severe end-organ damage after 20 weeks of gestation [4, 5] Preeclampsia impacts around 2–15% of pregnancies worldwide [6]. The association between race and preeclampsia frequency has traditionally been investigated in multicultural settings, comparing mostly Hispanic, African American, and White populations in the United States [7, 8], Sweden, and China [4]. Multifactorial diseases are attributed to both qualitative and quantitative changes from gene product variants, environmental, and incidental factors, with clinical phenotypes being manifested when these factors’ total effects exceed a certain threshold [9]. Early family-based research suggests that preeclampsia might be caused by a complicated mix of maternal, fetal, and paternal genetic factors [10, 11].

When PE develops without medical care, it can progress to eclampsia, which remains one of the top reasons for maternal and newborn death. It occurs solely in an instance of a placenta, even in the absence of an embryo (hydatidiform mole), and usually improves postpartum [12]. It should be highlighted that preeclampsia can be triggered by either hypoxia or fibrinolysis. Exposure to inflammatory cytokines such as VEGF and IL-1β increases the expression of plasminogen activator inhibitor-1 (PAI-1) in plasma [13]. Hypoxia can increase PAI-1 mRNA and protein expression by activating transcription factors, including hypoxia-inducible factors (HIF-1α) and HIF-2α [14]. In preeclampsia, high levels of syncytial PAI-1 can reduce maternal-to-fetal nutrition flow and hence trigger intrauterine growth restriction [15].

The PAI-1 gene is situated on chromosome 7 q21.3-q22, comprises nine exons, and spans around 12.3 kb [16]. It is expressed in a variety of organs, with endothelial cells producing the highest circulating amounts of the protein [17]. PAI-1 protein suppresses the activation of plasminogen to plasmin through inhibition of tissue plasminogen activator, and it is well known that PAI-1 downregulates the fibrinolytic system and, subsequently, upregulates the coagulation pathway and inflammatory reactions [18]. High heritability values for circulating levels of the protein have been found, indicating that common variations of the PAI-1 gene could be involved in gene expression regulation. Several gene variations have been linked to PE; approximately one hundred polymorphisms of the gene were discovered, but only a few of them have been studied in PE and other conditions. However, there is a lack of consistency among candidate genetic association studies [19]. There is still a dispute over the link among preeclampsia and PAI-1 mutations. PAI-1 (rs1799889) represents an insertion/deletion variation in the SERPINE1 promoter area, commonly referred to as PAI-1 5G/4G. The 5G allele refers to a sequence of 5 guanine nucleotides at − 675 of the PAI-1 promoter. The “4G” allele results from the deletion of one nucleotide [20]. Several studies have found that the polymorphism − 675 4G/5G has a role in the variance of PAI-1 plasma levels [21, 22]. It should be noted, however, that this mutation has different effects on different races, emphasizing the importance of examining it in Egyptian society. The current study was conducted to evaluate the role of the − 657 4G/5G PAI-1 polymorphism in pre-eclampsia patients in order to assess its role in influencing the onset of pre-eclampsia and its link to many pregnancy-related pathological problems, such as hypertension and elevated hepatic enzymes, in addition to elevated levels of oxidative stress in Egyptian women.

## Materials and methods

This case-control study was performed on 140 women with a diagnosis of PE in accordance with the International Association for the Studies of Hypertension definition (blood pressure > 140/90 mmHg and proteinuria > 0.3 g in 24-hour urine samples after 20 weeks of pregnancy) in the case group and 100 healthy pregnancies in the control group [5]. The samples were collected from October 2021 to August 2022 at Mansoura University Hospital from the Department of Obstetrics and Gynecology according to the ethical standards of the Institutional Research Board (IRB), Faculty of Medicine, Mansoura University (IRB: R.21.09.1447, date: 10/10/2021). Each participant signed the consent form. The study excluded participants with medical conditions related to pregnancy, whether previous or current.

### Blood sampling

Five milliliters of blood were collected via vein puncture from all participants. Each collected blood sample was either dispensed into EDTA tubes for molecular studies or allowed to collect serum after centrifugation for biochemical parameter measurement. All samples were obtained and then stored at -20 °C. Prior to the procedure, they had been left to be stored at room temperature.

### Extraction of DNA and genotyping

The genomic DNA was extracted from peripheral blood cells according to the Thermal Cycler TECHEN TC-312 using the DNA Purification Blood Mini Kit supplied by Qiagen GmbH, Cat. No. 51,104, Hiden, Germany. The purified DNA was used immediately in the PCR application. Evaluation of the − 657 4G/5G PAI-1 polymorphism was done using an amplification refractory mutation system-polymerase chain reaction (ARMS-PCR) [23]. The primers to be used are as follows: CCTAAAAGCACACCCTGCAA for the common primer; 5G allele: -ACACGGCTGACTCCCCCA-3 and 4G: 5’-ATACACGGCTGACTCCCCA-3`. The predicted product size was 312 bp for the 5G allele and 313 bp for the 4G allele. Two tubes were used for every subject. Each mixture of the PCR process was inserted in a 30 µl total volume containing 5 µl of common primer, 15 µl of master mix (fermentase), and 5 µl of DNA in a small PCR tube. This mix was inserted into 5 µL of definite primer, 4G primer, or 5G primer in distinct tubes. PCR products were performed at the final holding temperature of 4 °C. The PCR samples were amplified in a Gradient Thermo cycle Eppendorf, employing a T professional thermocycler (Biometra, Germany). The PCR procedure conditions were: primary denaturation at 95 °C, 4 min for 1 round; 33 cycles including denaturation at 95 °C, 25 s; then annealing at 60 °C, 30 s; extension step at 72 °C, 25 s; final extension at 72 °C, 10 min for 1 cycle; and then soaking at 4 °C. The products of PCR were separated on an agarose gel with 2.5% concentration, stained with ethidium bromide stain, and visualized via UV transillumination.

### Biochemical parameters

Serum ALT, AST, albumin, creatinine, and glucose were given to all participants according to the manufacturer’s protocols by an automatic analyzer (Hitachi 801) utilizing commercially available kits: serum albumin (BioMed, Cat. No. ALB100250), ALT (Linear Chemicals, Cat. No. 1105000), AST (Linear Chemicals, Cat. No. 1109010), creatinine level (Cat. No. CRE106100), and random blood glucose level (Spin React, Spain). A complete blood count (CBC) was done as a regular analysis by the Coulter Counter (MEK-631 K, Kohden Co., Japan) system. Lipid peroxidation was determined in terms of malondialdehyde (MDA) (nmol/mL) [24] and superoxide dismutase activity (SOD) (U/mL) [25]. Glutathione (GSH) was estimated using bio diagnostic colorimetry and expressed in ng/mL [26]. Levels of GPx were measured using the Bio-diagnostic Company (Egypt)TM enzymatic assay for cellular glutathione peroxidase, expressed as U/mL [27].

### Statistical analysis

SPSS package software (Armonk, New York) was used to analyze the data (Version 25). Continuous variables are reported as mean ± SD and discrete variables as percent. *P* < 0.05 was deemed statistically significant. The Kolmogorov–Smirnov test was performed for the distribution of continuous data (age, BP, biochemical parameters). Chi-square and Fisher’s Pearson’s chi-square tests were used to evaluate the genotype distribution of mutations, as well as the frequency of homozygous and heterozygous states, between patients and the control group. The one-way ANOVA approach was used to compare genotypes and parameters between group [28, 29].

## Results

The baseline characteristics of preeclamptic patients and controls in the study cohort are described in Table 1. The patients with preeclampsia had a higher mean ± SD age (28.96 ± 6.9 years) compared to healthy pregnant women (25.6 ± 4.2 years). The study compared blood pressure measurements and laboratory results between sufferers and healthy participants. PE patients showed considerably higher SBP (*P* < 0.0001), ALT (*P* < 0.009), and RBG (*P* < 0.0001) than controls. PE patients exhibited substantially lower HB (*P* < 0.0001), platelet count (*P* = 0.002), and albumin (*P* < 0.0001) compared to the normotensive pregnant. Serum creatinine was lower in patients than controls but did not give a significant value (*P* = 0.12). A highly statistically significant increase was found in the activity of GPx and the level of MDA in PE patients compared to controls (*p* < 0.0001 and 0.03, respectively). while the SOD activity and GSH level decreased significantly in PE patients compared to healthy subjects (*P* < 0.0001).

**Table 1:** Comparison of clinical and biochemical laboratory data among studied groups

Parameters	Patients group (*n* = 140) Mean ± SD	Control group (*n* = 100) Mean ± SD	*P*
**Age (year)**	28.96 ± 6.9	25.6 ± 4.2	**< 0.0001**
**SBP (mmHg)**	151.4 ± 18	124 ± 4.2	**< 0.0001**
**DBP (mmHg)**	92.6 ± 12	82 ± 3	**< 0.0001**
**HB (g %)**	10.38 ± 1.4	12.31 ± 1.25	**< 0.0001**
**PLT (K/µL)**	189.5 ± 27.7	215 ± 35	**0.002**
**albumin (g/dL)**	3.025 ± 0.6	3.9 ± 0.3	**< 0.0001**
**AST (IU/L)**	32.4 ± 5.1	29.38 ± 9	0.2
**ALT (IU/L)**	29 ± 3.4	21 ± 8.6	**0.009**
**RBG (mmol/L)**	109 ± 35	86 ± 4.9	**< 0.0001**
**Creatinine (mg/dL)**	0.782 ± 0.14	0.815 ± 0.19	0.12
**SOD (U/mL)**	89.18 ± 19.7	243.5 ± 23.15	**< 0.0001**
**GSH (ng/mL)**	0.11 ± 0.08	1.73 ± 0.365	**< 0.0001**
**MDA (nmol/mL)**	0.55 ± 0.11	0.49 ± 0.25	**0.03**
**GPx (U/mL)**	498.56 ± 53.3	303.22 ± 71.59	**< 0.0001**

Data were expressed as Mean ± SD; P, comparison between control and PE; *P* ≤ 0.05, significant; SBP and DBP systolic and diastolic blood pressure; HB, hemoglobin; PLT, platelet count; AST, aspartate transaminase; ALT; alanine transaminase; RBG, random blood glucose; SOD, superoxide dismutase; GSH, glutathione; MDA, malondialdehyde; GPx, glutathione peroxidase, Kolmogorov–Smirnov and t-test were used

### Distribution of PAI-1 4G/5G gene polymorphisms in PE cases compared to controls

**Fig. 1:**
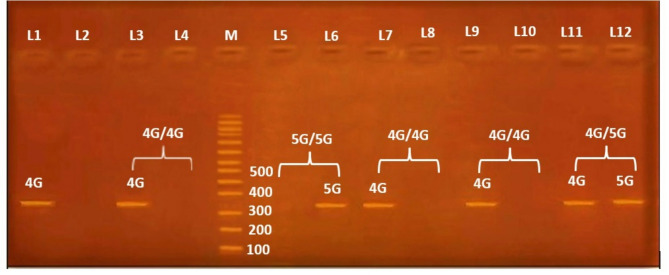
Electrophoretic pattern of PAI-1-675 4G/5G PCR product by ARMS PCR; M represents 100 bp DNA marker. Each of the two lanes represents one sample. Lanes 1, 2, 3, 4, 7, 8, 9, and 10 represent 4G/4G homozygous genotyping, where the 4G allele appears in lanes 1, 3, 7, and 9 at 313 bp and the 5G allele is absent from lanes 2, 4, 8, and 10. Lanes 5 and 6 represent 5G/5G homozygote genotyping, where the 4G allele is absent in lane 5 while the 5G allele is present in lane 6 at 312 bp. Lanes 11 and 12 represents 4G/5G heterozygote genotyping, with the 4G allele present in lane 11 at 313 bp and the 5G allele present in lane 12 at 312 bp

The PAI-1 4G/5G gene variations were analyzed in 100 PE patients as well as 100 healthy controls. The study revealed no significant divergence from Hardy-Weinberg equilibrium, implying that the genotype and allele rates of SNP in the population are unlikely to vary in subsequent generations. The homozygous 4G/4G in PE patients (7%) increased compared to that in normal healthy women (4%), while the heterozygous 4G/5G (31%) in patients was reduced compared to that of healthy subjects (66%). The homozygous 5G/5G genotype was greater in patients than in normal subjects. Table (2) shows a significant distinction between the patients’ group as well as the control group regarding PAI-1 (rs1799889) in the co-dominant model (*P* < 0.0001, OR = 2.1). Furthermore, there is a significant difference between the dominant (*P* = 0.004, OR = 2.45) and the recessive model (*P* = 0.009, OR = 1.8). Conversely, the over dominant model yielded no significant results (*P* = 0.08; OR = 0.99). According to allelic models, the findings demonstrated that there is a highly significant distinction between healthy and pregnant patients (*P* = 0.004, OR = 1.22) (Fig. 1).

**Table 2:** The correlation between PAI-1 (rs1799889) gene polymorphism among patients and healthy subjects:

Model	Genotype	Patients (*n* = 100)		Controls (*n* = 100)		OR (95%CI)	*P*-value
		*n*	%	*n*	%		
**general**	**5G/5G**	62	62	30	30	2.1 (1.1–4.5)	**< 0.0001**
	**4G/5G**	31	31	66	66		
	**4G/4G**	7	7	4	4		
**Dominant**	**5G/5G Vs.** **4G/5G-4G/4G**	62	62	30	30	2.45 (1.1–4.5)	**0.004**
		38	38	70	70		
**Recessive**	**4G/4G Vs.** **4G/5G-5G/5G**	7	7	4	4	1.88 (1.1–3.89)	**0.009**
		93	93	96	96		
**Over-dominant**	**4G/5G Vs.** **4G/4G-5G/5G**	31	31	66	66	0.99 (0.76–2.1)	0.08
		69	69	31	31		
**Alleles**	**5G**	155	77.5	126	63	1.22 (1.5–3.5)	**0.004**
	4G	45	22.5	74	37		

P, probability; *P* ≤ 0.05, significant; OR, Odds ratio; CI, confidence interval

### The associations between PAI-1 (rs1799889) and investigated parameters within the studied groups

In order to clarify the clinical implications of the PAI-1 4G/5G variation, we analyzed clinical features of participants with different genotypes across cohorts. Table 3 reveals that the 4G/4G genotype has a considerably stronger connection with SBP and DBP than the 4G/5G and 5G/5G groups (*P* = 0.01, *p* = 0.023, respectively). When compared to homozygous 4G or 5G, the 4G/5G genotype had a significantly higher hemoglobin level (*P* = 0.001). Serum albumin and RBG exhibit a significant relationship in the studied group with 4G/5G and 5G/5G, respectively. There was no significant link seen between AST, ALT, platelet count, or creatinine levels within groups.

**Table 3:** Association of PAI-1 -675 4G/5G with laboratory parameters within groups

Clinical parameters	PAI-1 Genotype (rs1800896)							One-way ANOVA
	4G/4G		4G/5G		5G/5G			
	no	Mean ± SD	no	Mean ± SD	no	Mean ± SD	F	*P* value
**Age (Year)**	11	28.3 **±** 5.3	97	26.6 **±** 5.6	91	27.4 **±** 6	1.78	0.1
**SBP (mmHg)**	11	141.3 **±** 19	97	133 **±** 16	91	141.6 **±** 22	4.6	**0.01**
**DBP (mmHg)**	11	89.4 **±** 10.9	97	85.4 **±** 9.4	91	89.8 **±** 11	3.8	**0.023**
**HB g%**	11	11.5 ± 1.3	97	11.8 ± 1.4	91	10.9 ± 1.7	6.8	**0.001**
**Platelet count (K/ µL)**	11	170.9 ± 25	97	208.7 ± 27	90	199.4 ± 36	2.1	0.1
**ALT (IU/L)**	11	21.9 ± 4.2	92	28.2 ± 3.2	87	23.5 ± 1.2	0.9	0.3
**AST (IU/L)**	11	36.9 ± 2.8	93	31.4 ± 1.8	86	30.5 ± 1.8	0.5	0.5
**Serum albumin (g/dL)**	11	3.2 ± 0.5	91	3.7 ± 0.6	78	3.3 ± 0.6	9	**< 0.0001**
**Creatinine (mg/dL)**	10	0.75 ± 0.08	92	0.78 ± 0.1	82	0.81 ± 0.1	1	0.3
**RBG (mmol/L)**	9	109.5 ± 28	85	90.8 ± 21	74	95 ± 20	3.4	**0.03**

SBP, systolic blood pressure; DBP, Diastolic blood pressure; HB, Hemoglobin; ALT, alanine transaminase; AST, aspartate transaminase; RBG, blood glucose; P, probability; *P* ≤ 0.05, significant; Data were expressed as Mean ± SD. Results were obtained using the One-Way ANOVA test

Oxidative stress measures significantly increased in the 4G/5G heterozygous genotype for SOD and GSH among subjects (*P* = 0.002, *p* < 0.0001, respectively). There was a considerable rise in the 5G/5G homozygous genotype with GPx among pregnant women (*P* = 0.021). There is no significant difference between PAI-1 genotypes and MDA levels within all pregnant women (*P* = 0.2). Table 4.

**Table 4:** Association of PAI-1 -675 4G/5G with the oxidative stress parameters within subjects

Oxidative stress parameters	PAI-1 Genotype			Test of significance	*P*
	**4G/4G(n =** 11)	**4G/5G(n =** 94)	**5G/5G(n =** 94)		
	Mean ± SD	Mean ± SD	Mean ± SD		
**SOD (U/mL)**	178.4 ± 36	**192 ± 30**	142.1 ± 18	F = 6.3	**0.002**
**GSH (ng/mL)**	0.67 ± 0.08	**1.2 ± 0.3**	0.66 ± 0.02	F = 10.5	**< 0.0001**
**MDA (nmole/mL)**	0.51 ± 0.02	0.49 ± 0.05	0.55 ± 0.01	F = 1.2	0.2
**GPx (U/mL)**	365.7 ± 31	356.3 ± 46	451 ± 53	F = 3.9	**0.021**

MDA, Malondialdehyde; SOD, Superoxide dismutase; GSH, Glutathione; GPx, Glutathione peroxidase; P, probability; *P* ≤ 0.05, significant; Data were expressed as Mean ± SD. Results were obtained using the One-Way ANOVA test

## Discussion

Pre-eclampsia is characterized by decreased blood flow to the placenta, which releases substances that stimulate the maternal vascular endothelium. According to research, women with pre-eclampsia have greater plasma levels of PAI-1 than gestationally matched pregnant women with no hypertension. However, it isn’t easy to discern between causative and epiphenomenal molecular pathways [30]To address this issue, researchers investigated whether genetic variant that increase PAI-1 expression is linked to the risk of pre-eclampsia. PAI-1-675G/A (4G/5G) has been investigated to be related to many diseases, such as ischemic stroke [31], type 2 diabetes mellitus [32], metabolic syndrome [33], and diabetic nephropathy [34].

The current research focuses on exploring the − 675 4G/5G PAI-1 gene variant relationship to preeclampsia. It seeks to assess the role of genetic variables, oxidative indicators, and antioxidants in preeclampsia. The insertion/deletion mutation at the SERPINE1 gene PAI-1 -675 (rs1799889) is a prevalent, well-characterized, and functionally significant single-nucleotide variant. The guanine deletion variation at position − 675 nucleotide related to the transcription start domain is the most extensively investigated functional variant in the PAI-1 gene [1, 2]. The 5G allele remains the major one, while the absence of one G nucleotide leads to the 4G minor allele, associated with greater plasma PAI-1 levels [35]. While both alleles can bind transcription activation, only the 5G allele enables the repressor protein to connect to an overlapping interaction site [36, 37].

The current study found an important distinction in PAI-1 rs1799889 genotype among preeclamptic women and controls in the general model (*P* < 0.0001). Also, there was a very significant statistical in both the dominant (5G/5G Vs. 4G/5G-4G/4G) and recessive (4G/4G Vs. 4G/5G-5G/5G) models (*P* = 0.004 and *P* = 0.009, respectively). Additionally, the allelic model shows a very substantial distinction between preeclamptic women and controls (*P* = 0.004). Our study demonstrated a substantial link between preeclampsia and a PAI-1 5G/5G genotype, contrary to previous research that linked preeclampsia to a PAI 4G/4G genotype and higher PAI-1 antigen levels [35]. In agreement with the current research, a case-control study of German women found that women who carry the PAI-1 5G/5G genotype are more likely to develop severe preeclampsia early [35]. The underlying pathophysiology of preeclampsia is believed to be a placental abnormality caused by trophoblast invasion failure [38]. Previous research has shown that decidual cells of the secretory phase and pregnant endometrium release two important hemostasis modulators: tissue factor, the major initiator of hemostasis via factor VIIa, and, in turn, factor Xa activation and PAI-1 [39]. The coordinated rise in tissue factor and PAI-1 expression explains how decidual cells regulate local hemostasis during endovascular trophoblast invasion [38, 40]. Thus, reduced PAI-1 antigen levels may be linked to impaired trophoblast invasion. This method could explain the deleterious influence of the PAI-1 5G/5G genotype, which is known to be associated with reduced PAI-1 antigen levels. S. Mütze et al.‘s findings revealed that preeclamptic study yielded conflicting results [41]. A preceding systematic review indicates that people with the 4G allele had greater plasma PAI-1 levels, which could contribute to the occurrence of pre-eclampsia [30]Also, a systematic review including six case-control studies with 880 patients and 810 healthy women has been evaluated to find that 54.9% of people with the 4G gene develop pre-eclampsia, compared to 43.1% of participants without the 4G allele. According to total risk estimates, people with 4G alleles are 1.27 times more likely to have pre-eclampsia [42]. In a meta-analysis of preeclamptic patients, investigating 11 studies involving 1297 PE cases and 1791 controls, the 4G allele contributes to an increased risk of preeclampsia [13]. Additionally, De Maat et al. investigated the frequencies of the various 4G/5G genotypes in preeclamptic and healthy pregnant women and discovered no significant variation in the distribution of frequencies between women with significant preeclampsia and healthy control women [43]. The findings of past and current investigations may differ due to changes in medical diagnostic procedures and ethnicity across the research groups.

With an alteration in the demographics of preeclampsia, older women now account for a growing share of preeclampsia and its concomitant unfavorable effects [44]. The incidence of preeclampsia in the general obstetric populace is 3–4%, rising to 5–10% in women over 40 and reaching 35% in those over 50 [31]. The current context reveals a highly significant age difference between patients and the control group, indicating that age is a crucial component in preeclampsia etiology. A Chinese study supports our findings by seeing enhanced un-favorable gestation impacts associated with high mother ages [32]. Increased prevalence of overweight and obese moms likely contributes to age-related differences in PE. When pertaining to the hypertension in the groups under study, the data currently available demonstrates that there is a substantial variance (*P* < 0.0001) in both the systolic and diastolic blood pressures between the patient and control groups. This difference highlights the hypertension that is particularly prevalent during the severe stages of the PE disease. Similar findings with noticeably increased BP were observed by another big investigation with one thousand patients with preeclampsia and pregnancy hypertension [33]. Another cross-sectional study included 55 women with preeclampsia who saw a steady rise in BP from the control group to those with gestational hypertension [34]. Studies done in Yemen [45], Ethiopia [46], and Jordan [47] indicated that hypertension increases the risk of PE by 14 times. The PAI-1 gene polymorphism has been associated with elevated blood pressure in preeclampsia, with *P* = 0.01 and 0.023 for SBP and DBP, respectively. A higher PAI-1 level was associated with a 35% increased chance of developing hypertension, indicating that it may contribute to hypertension development in addition to established risk factors [48]. Anemia, urinary tract infections, and gestational diabetes mellitus are a few risk factors linked to pregnancy-related hypertension [49]. Anemia is a widespread disorder, particularly in developed countries. Hematologic abnormalities such as thrombocytopenia and low HB levels are among the most common disorders that can be detected with a conventional CBC test in PE [50]. Our results show a substantial (*P* < 0.0001) difference in anemia HB among patients when compared to the control group. In contrast to our findings, another published study revealed that anemia during the first 6 months of gestation did not correlate with PE [51]. The mean platelet levels were lower in the patient group and higher in the control group, with a significant difference (*P* = 0.002). A previous study that contrasted with our findings suggested that thrombocytopenia might be a late lab indication of PE, and declining concentrations are generally accompanied by more acute disturbance [52]. Research indicates that the count of platelets during the first three months of gestation is not a reliable indicator for predicting PE incidence [53].

Determining liver enzymes can be extremely valuable in preventing the harmful repercussions of PE. Insufficient blood flow to the liver can cause hepatic chemistry defects, potentially resulting in per-portal hemorrhage and ischemia [54]. The current study found a significant variance in ALT between the patient and control groups (*P* = 0.009). Despite the presence of elevated levels of AST in patients, suggesting that ALT may be a key risk factor for PE. The study found a statistically significant difference in mean albumin levels among the participants (*P* < 0.0001). Our findings were consistent with numerous research [55–57]. A different investigation discovered that the blood albumin level in the PE group was the same as that of the normotensive group, contradicting our results [58]. PE as well as gestational diabetes (GDM) share multiple pathways. A study found that a history of preeclampsia might be a further risk factor for GDM. Insulin resistance or elevation can cause excessive sympathetic activity, inappropriate salt absorption, endothelial cell damage, and an increased risk of PE [59]. Our study found a substantial difference in RBG levels between participants (*P* < 0.0001), suggesting that RBG may be a key risk factor for PE. Retrospective research discovered no independent association between GDM and prenatal diabetes [60]. There was an important distinction in RBG with the PAI-1 gene polymorphism (*P* = 0.03). Research discovered a substantial difference in RBG with the PAI-1 gene polymorphism between PE patients and healthy subjects [61]. However, another study found that PAI-1 levels in maternal uterine blood were unchanged in GDM women during the third trimester of pregnancy [62].

Oxidative damage contributes to PE pathophysiology. Several studies have found that females with PE have higher amounts of superoxide in their placenta tissues compared to normal pregnant women [63]. Oxidative stress is more prevalent in healthy pregnant women, but activated free radicals raise PE levels, causing muscular tension and increased vascular tone. It is uncertain whether antioxidant deficiencies and oxidative stress are the primary causes or byproducts of PE [64]. A lack of antioxidant defenses can greatly enhance lipid peroxidation in PE. Elevated MDA levels in the current study suggest that lipid peroxidation may play a role in the pathophysiology of preeclampsia, revealing that preeclamptic females have much higher levels of circulating MDA than normal pregnant women, with a statistically significant difference (*P* = 0.03). Preeclamptic females were found to have an abnormally high level of biomarkers of oxidative stress and lipid peroxidation, which supports our findings [65]. A considerable loss in the total capacity of antioxidants was identified, in spite of the substantial elevation in overall peroxide as well as the final products for oxidative stress. Several studies have found that preeclamptic females have a much higher antioxidant capacity, whereas others have found a reduced capacity. The study found minimal SOD activity in preeclamptic females, with a significant result. This This is consistent with previous studies indicating lower SOD activity in preeclamptic females [66]. This decrease is related with increasing oxidative stress and lipid peroxidation, implying that SOD is consumed or its function hindered as preeclampsia progresses [67, 68]. Elevated DBP in preeclamptic females is associated with vascular damage leading to lipid peroxidation [69]. GSH, a critical lipid-suppressing tri-peptide, was discovered to be considerably lower in preeclamptic females than in normal pregnant women (*P* < 0.0001). This decline might be attributed to increased local or systemic oxidative damage. Its low levels might suggest considerably higher amounts of circulating hydrogen peroxide in PE, ensuring the importance of GSH in avoiding oxidative damage in preeclamptic women [70]. Our study found a substantial increase in GPx activity in preeclamptic females compared to normal pregnant females (*P* < 0.0001). Similar to the present outcome, PE’s enhanced GPx activity may work as a protective mechanism against damage produced by reactive radicals and other toxins. Regardless, the presence of GPx in preeclamptic females’ plasma might represent a defensive reaction to oxidative stress [71]. PAI-1 is implicated in mitochondrial malfunction, which is a contributing factor to oxidative stress [72]. However, the intricate relationships between PAI-1 and oxidative indicators, as well as the direct impact on oxidative stress levels, have not been confirmed. Further research is needed to properly understand these linkages. Among all studied pregnant women, there is a strong statistically significant (*P* = 0.0001) difference in GSH with PAI-1 gene polymorphisms all over the pregnant women regarding 5G/4G heterozygous genotype, a highly statistically significant (*P* = 0.002) difference in SOD with PAI-1 gene polymorphism regarding 5G/4G heterozygous, and a statistically significant (*P* = 0.021) difference in GPx with PAI-1 gene polymorphism regrading 5G/5G homozygous genotype also found, but there is no significant (*P* = 0.2) difference in MDA with PAI-1 gene polymorphism in the subjects.

In conclusion, these findings shed light on the genetic and biochemical factors contributing to the pathogenesis of pre-eclampsia in the Egyptian population. The study shows a strong association between the − 657 4G/5G PAI-I genotype and preeclampsia among Egyptian women. Furthermore, preeclampsia patients have lower levels of antioxidants like SOD and GSH and greater amounts of oxidative stress indicators like MDA and GPx than healthy people. While it is noteworthy that genetic variations were discovered between the preeclampsia and control groups, the small sample size, particularly among the control samples, is a problem that should be addressed in future research. Furthermore, the lack of functional research and information concerning the week of diagnosis and sample, as well as the inability to classify individuals as early or late-PE to enable further exploration to better understand the molecular mechanisms underlying these genetic discrepancies. Overall, the findings lay the groundwork for future research, but bigger sample numbers and functional investigations are required to reach clearer conclusions.

## Data Availability

Data is provided within the manuscript.
